# Supplementary material for: Integrins Can Act as Suppressors of Ras-Mediated Oncogenesis in the Drosophila Wing Disc Epithelium
Source: Cancers (Basel). 2023 Nov 15;15(22):5432. doi: 10.3390/cancers15225432 (PMC10670217; doi:10.3390/cancers15225432)
Supplement: Supplementary file 1 [file cancers-15-05432-s001.zip › Supplementary Figure and Movie Legends.pdf]

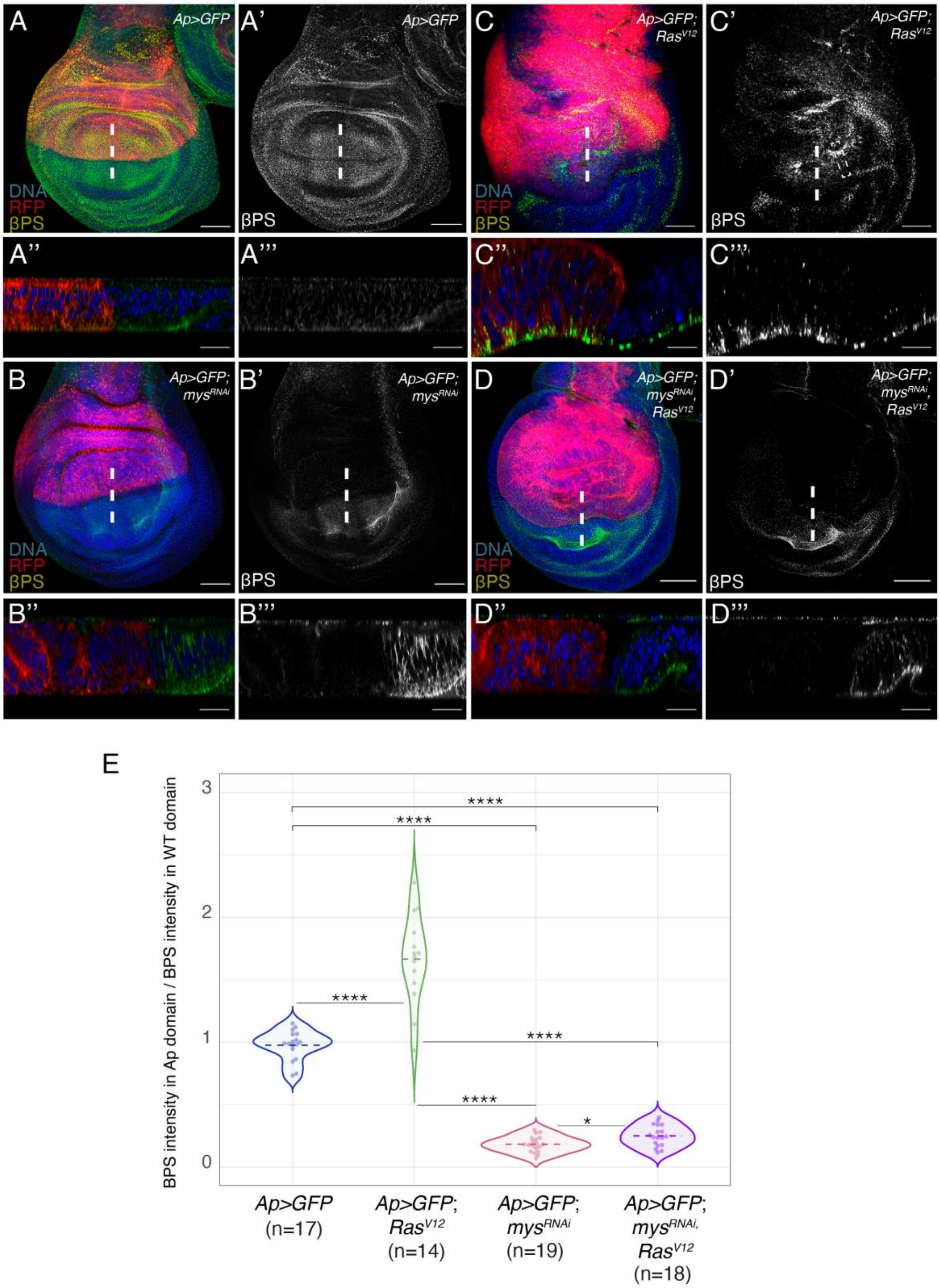

Figure S1. Oncogenic *Ras^{V12}* increase integrin expression levels in *Drosophila* wing disc epithelial cells. (A-D) Maximal projection of confocal views of third-instar larvae wing discs of the indicated genotypes, stained with anti-RFP (red), anti-βPS (green in A-D and A′′-D′′, white in A′-D′ and A′′′-D′′′) and Hoechst (DNA, blue). (A′′-D′′′) Confocal xz sections along the white dotted line shown in A-D′. (E) Violin plots of the intensity of βPS fluorescence in Apterous domain / intensity in the wild-type domain. The statistical significance of differences was assessed with a welch-test, ****, * P values are <0.0001 and <0.05, respectively. Scale bars 50 μm (A-D′) and 10 μm (A′′-D′′′).

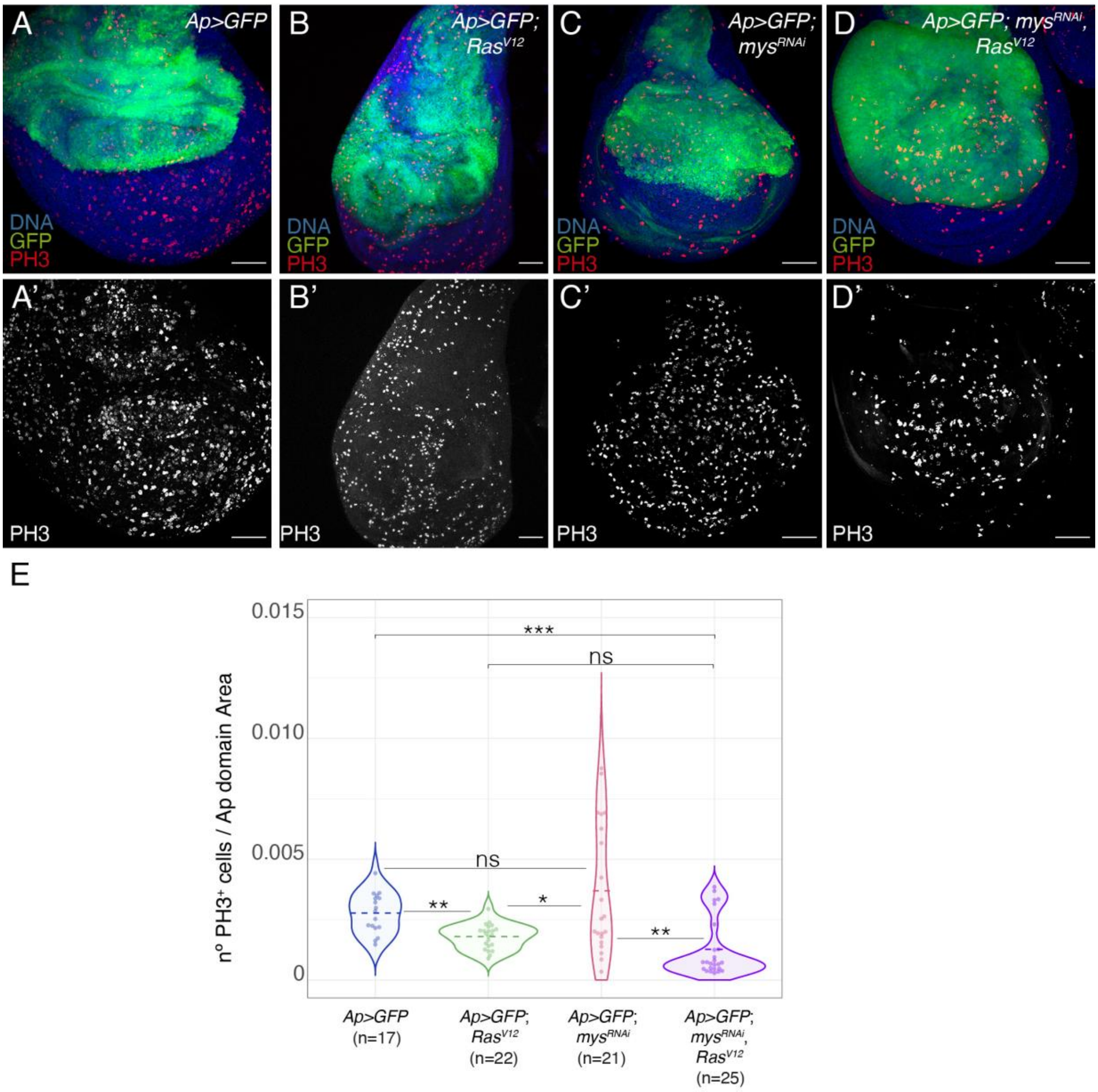

Figure S2. Downregulation of integrins does not affect the proliferation of *Ras^{V12}* tumoral cells. (A-D') Maximal projection of confocal images of third-instar wing discs of the indicated genotypes, stained with anti-GFP (green), antibody against PH3 (red) and Hoechst (DNA, blue). (A'-D') Same disc only stained with anti-PH3(grey). (E) Violin plots of number of $PH3^+$ cells/apterous domain area. The statistical significance of differences was assessed with a welch-test, ***, **, * P values are <0.001, <0.01 and <0.05 respectively. Scale bars are 40 µm.

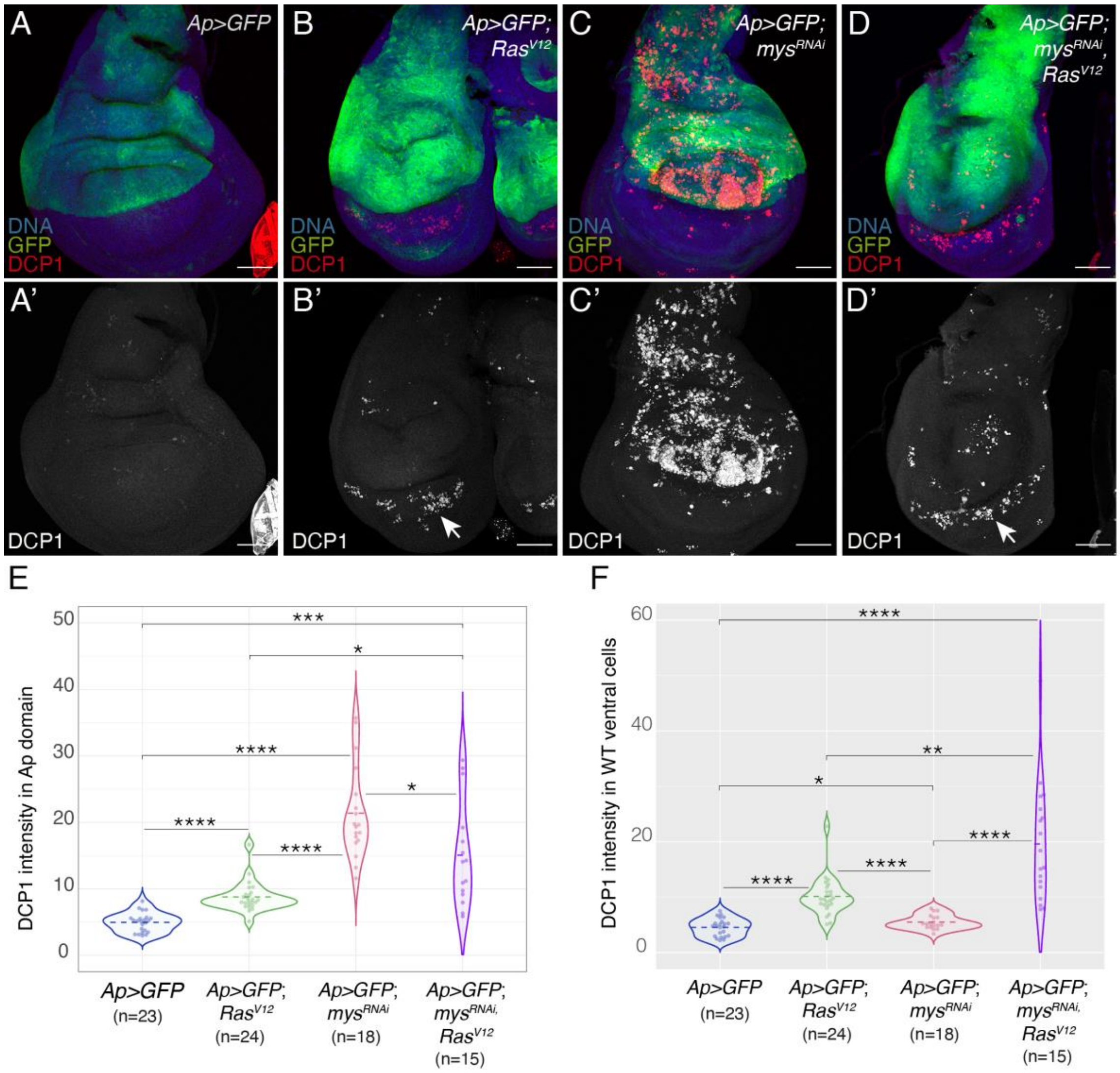

Figure S3. Downregulation of integrin expression increases the ability of *Ras*^{V12} cells to induce the death of nearby wild-type cells. (A-D) Maximal projection of confocal images of third-instar larvae wing discs of the specified genotypes, stained with anti-GFP (green), anti-DCP1 (red in A-D, white in A'-D') and Hoechst (DNA, blue). (E, F) Violin plots of the mean fluorescent DCP1 intensity in the apterous (E) and ventral (F) domains. The statistical significance of differences was assessed with a welch-test, ****, ***, **, * P values are <0.0001, <0.001, <0.01 and <0.05 respectively. Scale bars are 40 μm.

Movies S1-5. *In vivo* analysis of third instar cultured control *ap>GFP* (Movie 1), *ap>GFP*; *Ras*^{V12} (Movie S2), *ap*>GFP; *mys*^{RNAi} (Movie S3), *ap*>GFP; *mys*^{RNAi}; *Ras*^{V12} (Movie S4) and *ap*>GFP; *mys*^{RNAi}; *hid*^{RNAi} (Movie S5) wing discs.
