# Supplementary figures and images for: Integrins Can Act as Suppressors of Ras-Mediated Oncogenesis in the Drosophila Wing Disc Epithelium
Source: Cancers (Basel). 2023 Nov 15;15(22):5432. doi: 10.3390/cancers15225432 (PMC10670217; doi:10.3390/cancers15225432)

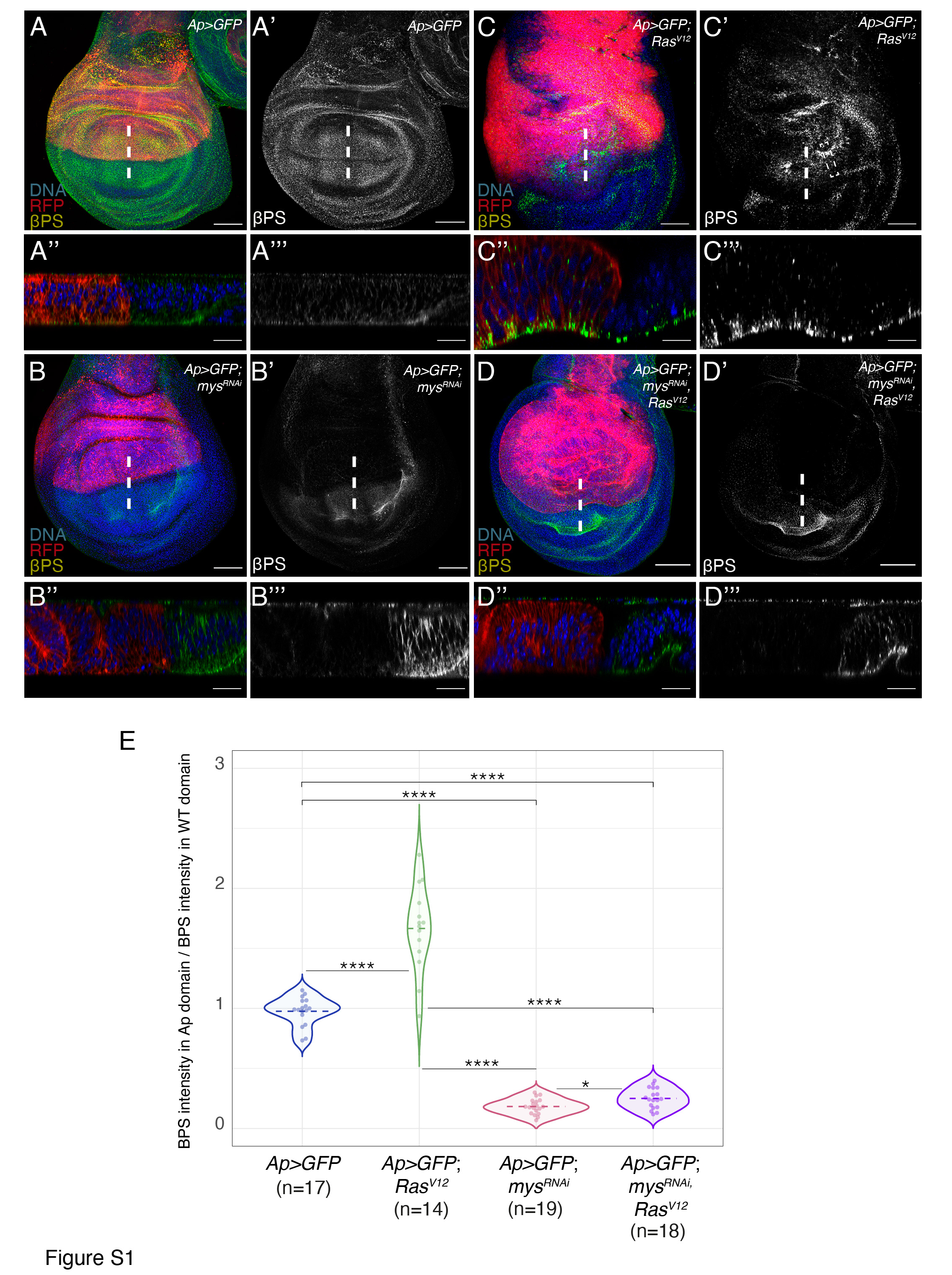

Supplement: Supplementary file 1 [file cancers-15-05432-s001.zip › Supplementary Figures/Sup. Fig.S1.jpg]

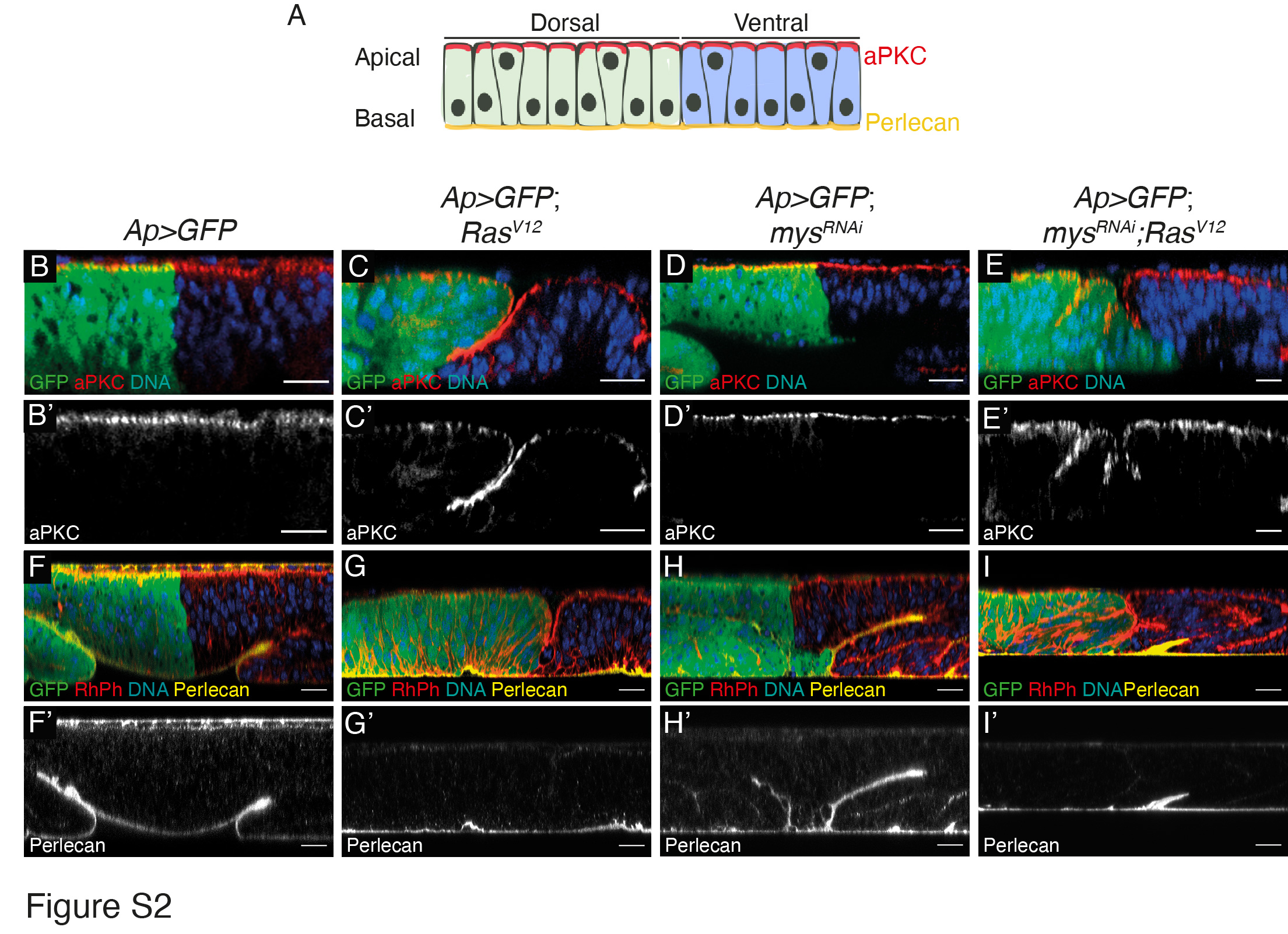

Supplement: Supplementary file 1 [file cancers-15-05432-s001.zip › Supplementary Figures/Sup. Fig.S2.jpg]

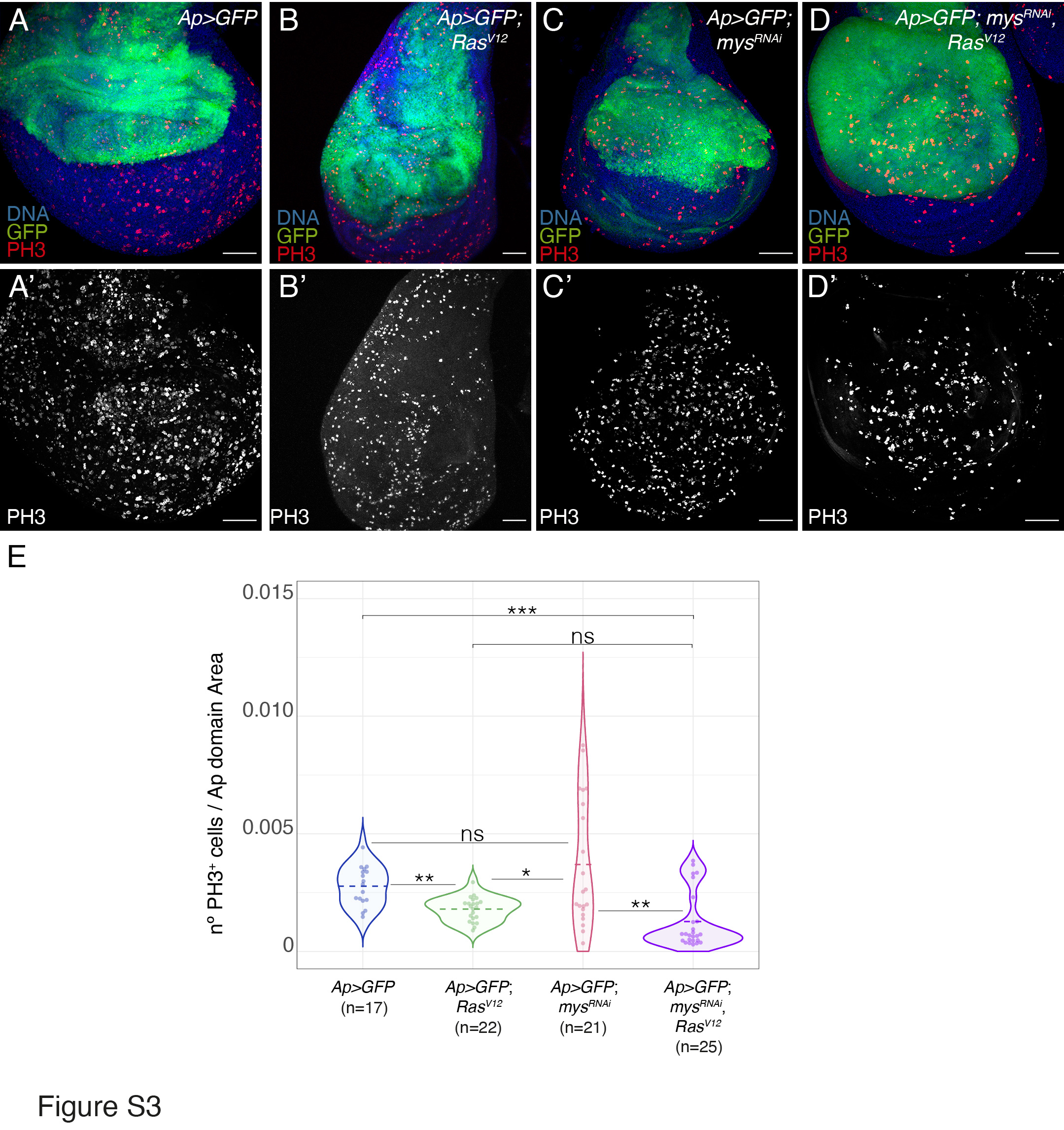

Supplement: Supplementary file 1 [file cancers-15-05432-s001.zip › Supplementary Figures/Sup. Fig.S3.jpg]

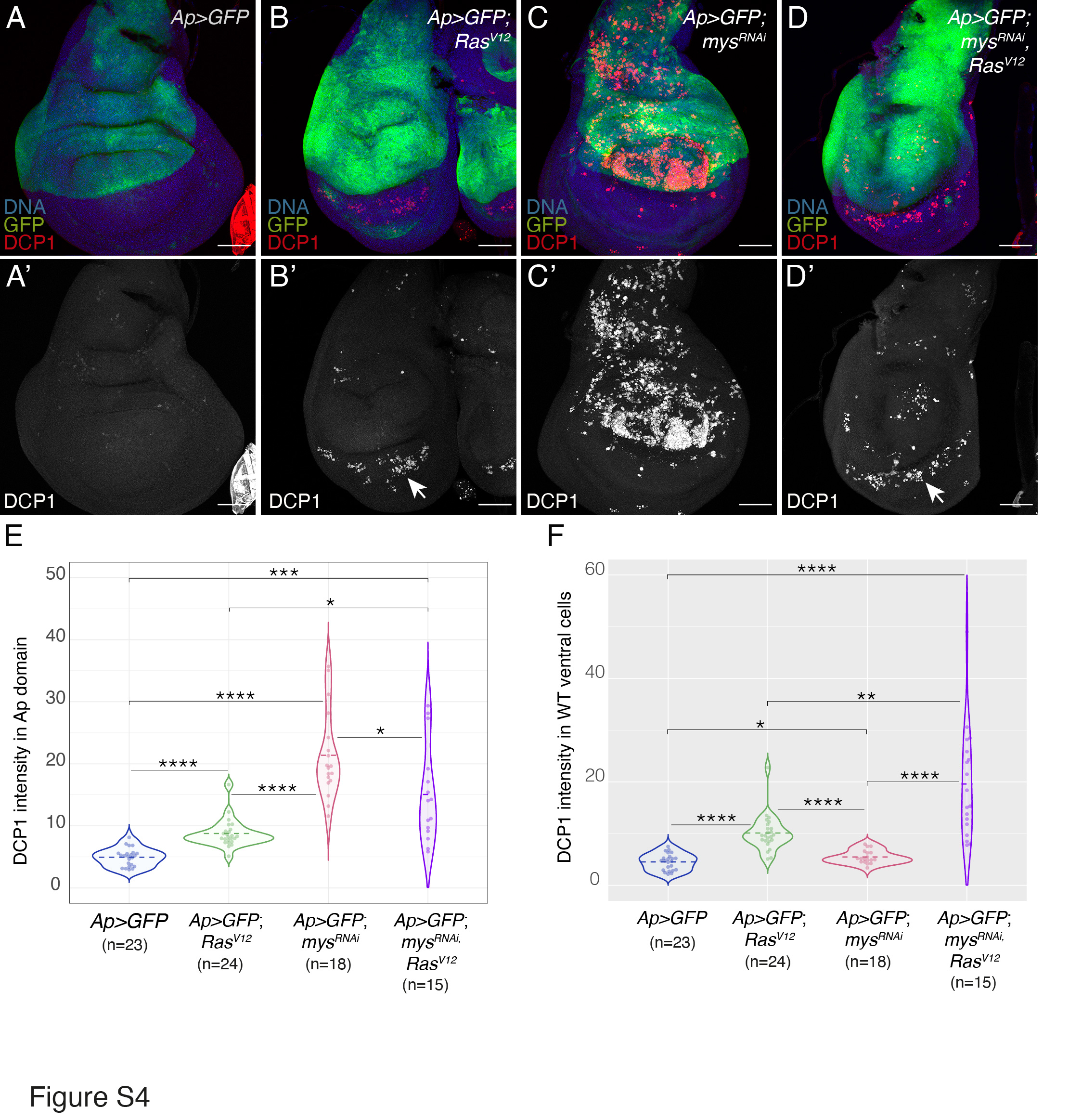

Supplement: Supplementary file 1 [file cancers-15-05432-s001.zip › Supplementary Figures/Sup. Fig.S4.jpg]
